# Quantitative analysis of mechanical force required for cell extrusion in zebrafish embryonic epithelia

**DOI:** 10.1242/bio.027847

**Published:** 2017-09-07

**Authors:** Sohei Yamada, Takanori Iino, Yasumasa Bessho, Yoichiroh Hosokawa, Takaaki Matsui

**Affiliations:** 1 Gene Regulation Research, Graduate School of Biological Sciences, Nara Institute of Science and Technology, 8916-5 Takayama-cho, Ikoma, Nara 630-0192, Japan; 2 Bio-Process Engineering, Graduate School of Materials Science, Nara Institute of Science and Technology, 8916-5 Takayama-cho, Ikoma, Nara 630-0192, Japan

**Keywords:** Femtosecond laser, Contractile ring, Contraction force, Enveloping layer

## Abstract

When cells in epithelial sheets are damaged by intrinsic or extrinsic causes, they are eliminated by extrusion from the sheet. Cell extrusion, which is required for maintenance of tissue integrity, is the consequence of contraction of actomyosin rings, as demonstrated by both molecular/cellular biological experimentation and numerical simulation. However, quantitative evaluation of actomyosin contraction has not been performed because of the lack of a suitable direct measurement system. In this study, we developed a new method using a femtosecond laser to quantify the contraction force of the actomyosin ring during cell extrusion in zebrafish embryonic epithelia. In this system, an epithelial cell in zebrafish embryo is first damaged by direct femtosecond laser irradiation. Next, a femtosecond laser-induced impulsive force is loaded onto the actomyosin ring, and the contraction force is quantified to be on the order of kPa as a unit of pressure. We found that cell extrusion was delayed when the contraction force was slightly attenuated, suggesting that a relatively small force is sufficient to drive cell extrusion. Thus, our method is suitable for the relative quantitative evaluation of mechanical dynamics in the process of cell extrusion, and in principle the method is applicable to similar phenomena in different tissues and organs of various species.

## Abstract (precis)

**Summary:** In this study a novel *in vivo* force quantification system was developed, which succeeded in estimating the magnitude of force required for extrusion of a dying cell from zebrafish embryonic epithelia.

## INTRODUCTION

The internal and external surfaces of the body are covered with epithelial sheets, and the primary role of these sheets is to provide a protective barrier against physical damage and infections. The integrity of the epithelial sheet is maintained throughout the lifespan of the organism (Madison, 2003), and turnover of epithelial cells occurs over short timescales (e.g. 5 days in intestine, and 26–28 days in skin) (Barker, 2014; Weinstein and Van Scott, 1965). If dying or dead cells remain in the epithelial sheet, epithelial integrity is disrupted, leading to disorders such as abnormal morphogenesis, inflammation, and cancer (Eisenhoffer et al., 2012; Igaki et al., 2009; Martin, 1997). Thus, organisms from invertebrates to mammals have evolved a system called cell extrusion, which removes dying/dead cells from the sheet without damaging neighboring healthy cells or disrupting barrier function (Eisenhoffer and Rosenblatt, 2013; Katoh and Fujita, 2012; Rosenblatt et al., 2001).

Previous studies reported critical roles for actin filaments and myosin motor proteins in the process of cell extrusion (Eisenhoffer and Rosenblatt, 2013; Katoh and Fujita, 2012; Rosenblatt et al., 2001; Tamada et al., 2007). When cell death in an epithelial cell is induced, filamentous actins (F-actins) in the surrounding cells accumulate, generating an actin ring. Subsequently, the Rho and Rho-associated kinase (ROCK) pathway is activated in the surrounding cells, leading to phosphorylation of myosin II and contraction of actomyosin (Kuipers et al., 2014; Rosenblatt et al., 2001; Tamada et al., 2007). As a result, the actomyosin ring shrinks at the basal side, pushing the dying cell out of the sheet. However, it remains unknown how much force is generated by the contraction of the actomyosin ring during cell extrusion, largely due to the lack of a system for direct measurement of such forces in epithelial sheets *in vivo*.

The mechanical forces generated by living cells have been measured *in vitro* for some biological processes, including cell migration and cell–cell or cell–matrix interactions. For instance, both traction force microscopy (TFM) and the micropillar assay track the deformation of synthetic elastic polymer substrates during cell migration, yielding an estimate of the magnitude of the traction force at leading edges of single cells during cell migration (Balaban et al., 2001; Discher et al., 2005; Fu et al., 2010; Munevar et al., 2001; Roca-Cusachs et al., 2017). In single-cell force spectroscopy (SCFS), varying magnitudes of tensional forces are loaded onto the interface between two cells, and the adhesive force between them is measured (Benoit et al., 2000; Krieg et al., 2008). However, these strategies require that the cells be removed from the organism and manipulated under specific experimental conditions that differ from the physiological environment. Therefore, such methods cannot measure the force generated in the process of cell extrusion, which occurs in the epithelial sheets of living organisms.

Previously, we developed a method using a femtosecond laser to generate impulsive force, allowing quantification of cell adhesion between cultured cells (e.g. leukocytes and endothelial cells), epithelial cells with one another, and neurons and mast cells (Hosokawa et al., 2011; Iino et al., 2016; Kaji et al., 2007). When a femtosecond laser is focused through an objective lens, a stress wave is generated at the laser focal point. The stress wave propagates spherically from the focal point, and acts to cells as an impulsive force. Consequently, when the laser is focused near a site of cell–cell contact, the adhesion is disrupted without damaging the cell. Furthermore, by measuring the magnitude of the impulsive force using an atomic force microscope (AFM), we succeeded in quantifying the force of cell adhesion *in vitro* (Hosokawa et al., 2011; Iino and Hosokawa, 2010).

In a recent study, we used the femtosecond laser to directly irradiate a specific type of cell within living zebrafish embryos, and succeeded in establishing a single-cell ablation technique *in vivo* (H.I., R.A., S.Y., Y.B., Y.H. and T.M., unpublished data). Our *in vivo* and *in vitro* results from that work inspired us to develop a new methodology for measuring the force generated by actomyosin ring contraction during cell extrusion from epithelial sheets in living organisms. In this study, we used the femtosecond laser as a loader of the external force, and the enveloping layer (an epithelial cell sheet of zebrafish embryos) as an *in vivo* model system for cell extrusion. Our results confirmed that force is generated by contraction of the actomyosin ring during cell extrusion. We also quantified the magnitude of the force by counter-balancing it with a calibrated impulsive force. Based on this measurement, we conclude that a relatively small force produced by actomyosin ring contraction is capable of driving cell extrusion.

## RESULTS

### Observation of dynamic changes in the actomyosin ring during cell extrusion in living zebrafish embryos

**Fig. 1.**
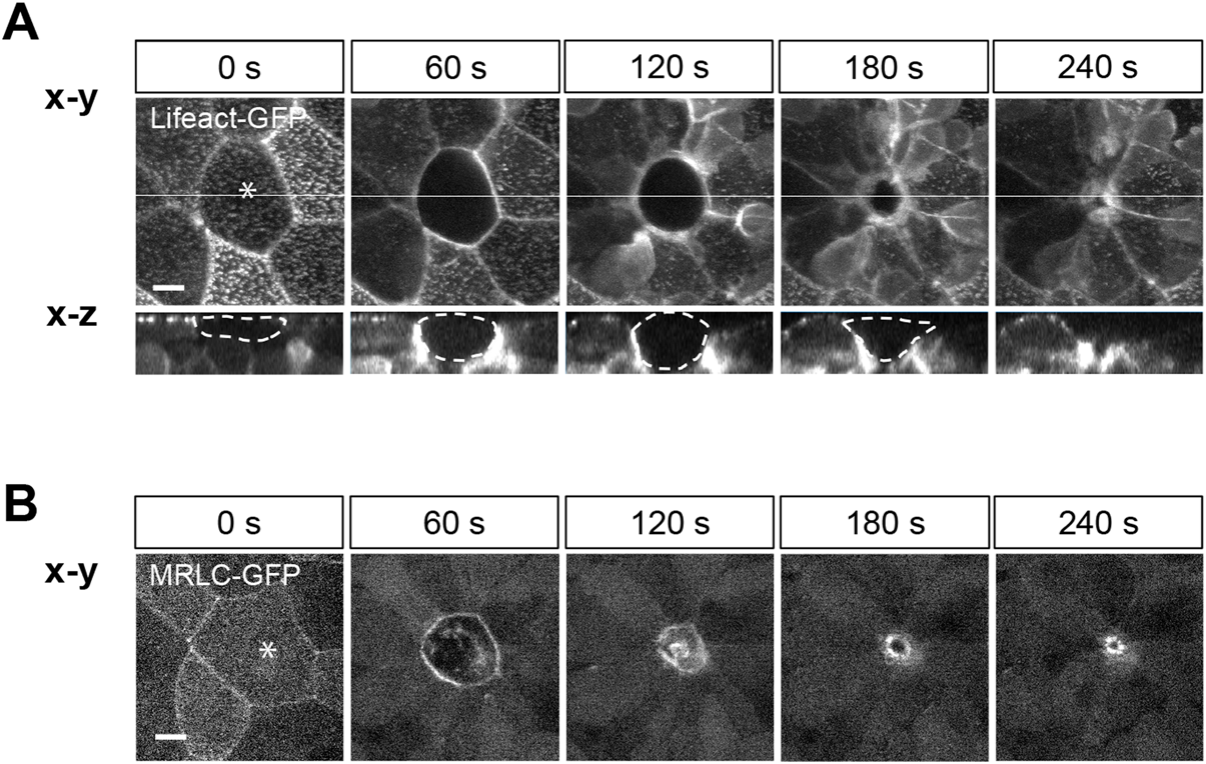
**Dynamics of actomyosin ring during cell extrusion in zebrafish embryonic epithelia.** (A) Dynamic changes of F-actin during cell extrusion in a Lifeact–GFP-overexpressing embryo. Representative images of cell extrusion were extracted from Movie 1. x-z views of images (lower panels) were obtained by the cross-section indicated by the white line in x-y views (upper panels). The targeted cell is marked by dotted white lines. (B) Dynamic changes of myosin II during cell extrusion in a MRLC–GFP-overexpressing embryo. Representative images of cell extrusion were extracted from Movie 2. At 0 s, the focal point marked by asterisks was irradiated with the femtosecond laser (in A and B). Scale bars: 10 µm.

First, we developed a system for observing the dynamics of the actomyosin ring during cell extrusion in living zebrafish embryos. To visualize the actomyosin ring, we overexpressed either Lifeact-GFP or MRLC-GFP, which labels F-actin or myosin II, respectively. In a parallel study, we established a single-cell ablation technique in living zebrafish embryos (H.I., R.A., S.Y., Y.B., Y.H. and T.M., unpublished data), and we used this method to induce cell death in epithelial sheets. When the center of an epithelial cell at mid-gastrulation stage was irradiated with the femtosecond laser, the dying cell was pushed out of the epithelial sheet (Fig. 1A). Both F-actin and myosin II accumulated at the membranes of surrounding cells within approximately 120 s after laser irradiation, leading to formation of an actomyosin ring (Fig. 1A,B; Movies 1 and 2). Subsequently, the actomyosin ring tightened until cell extrusion was complete (Fig. 1A,B; Movies 1 and 2). Thus, we succeeded in establishing a system for observing actomyosin dynamics during cell extrusion *in vivo*.

### Measurement of the mechanical force generated by contraction of the actomyosin ring during cell extrusion

**Fig. 2.**
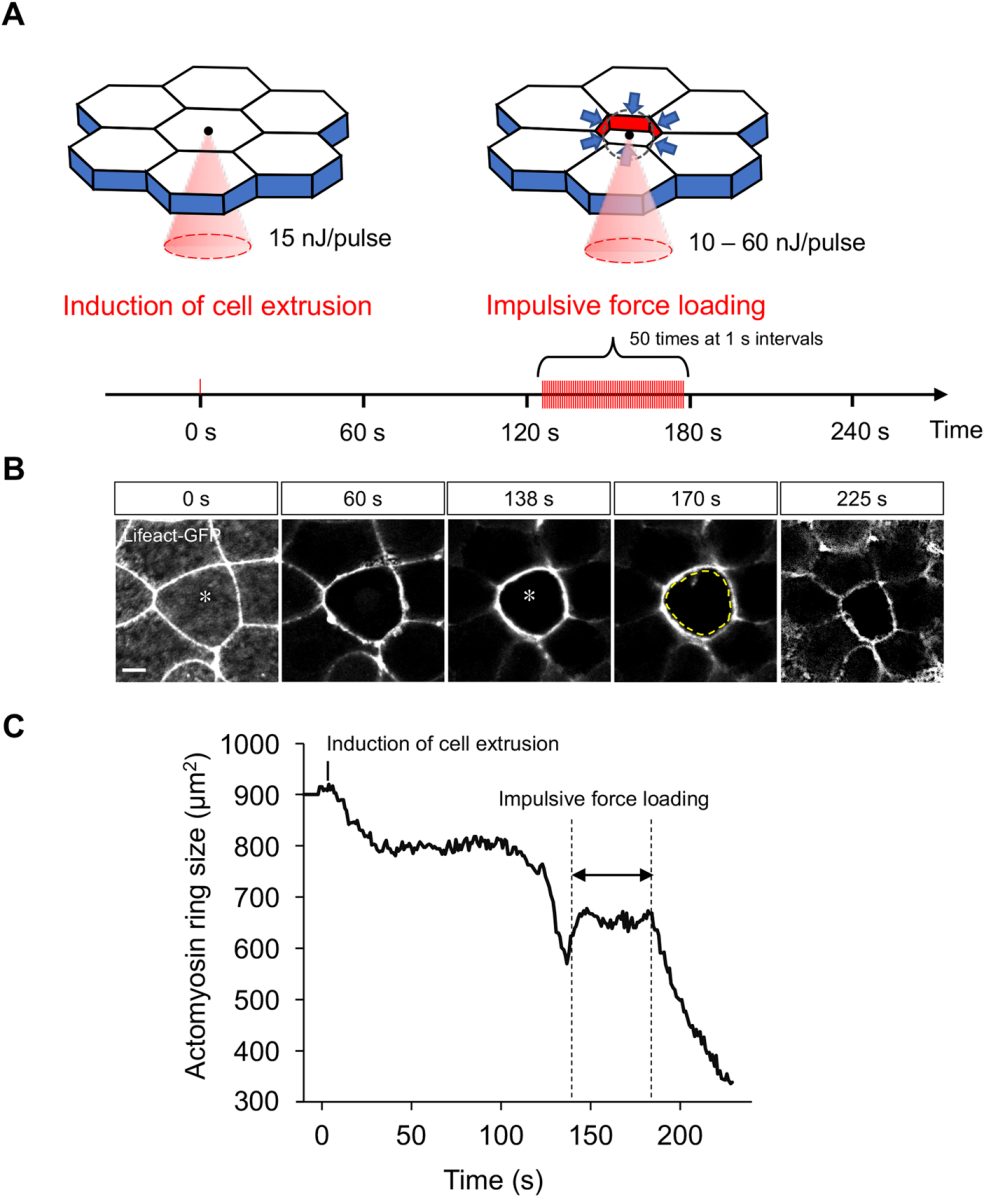
**Loading of impulsive force blocks tightening of the actomyosin ring during cell extrusion.** (A) Schematic of loading of impulsive force during cell extrusion. Left panel: To induce cell extrusion, the center of the targeted cell was irradiated with 15 nJ/pulse femtosecond laser at time 0 s (a red bar at time 0 s in lower panel). Right panel: After the actomyosin ring was generated and started to tighten, a series of laser pulses (10–60 nJ/pulse) was loaded every 1 s at the center of the actomyosin ring (red bars in lower panel). (B,C) Representative results of the impulsive force loading experiment in a Lifeact–GFP-overexpressing embryo. Still images (B) were extracted from Movie 3. Yellow dotted line in the panel at 170 s marks the size of the actomyosin ring at 138 s (before loading of impulsive force). Scale bar: 10 µm. Asterisks at 0 s and 138 s indicate the focal points of femtosecond laser. (C) To characterize actomyosin ring contraction, the size of the actomyosin ring was measured at each time point and plotted over time.

To measure the contractile force during cell extrusion, we first induced cell extrusion in zebrafish embryos via direct irradiation with the femtosecond laser (Fig. 2A, left panel, see also Fig. 1). Next, when the actomyosin ring was formed and started to contract (as noted above, approximately 120 s after laser irradiation), a series of impulsive forces was loaded (50 times at 1 s intervals) at the center of the actomyosin ring (Fig. 2A, right panel). At the beginning of force loading, the actomyosin ring did not contract, but instead expanded (Fig. 2B,C; Movie 3). After the expansion, contraction was interrupted during the period of force loading (Fig. 2B,C; Movie 3); however, when force loading was stopped, the ring contraction restarted (Fig. 2B,C; Movie 3). This observation suggests that, when the impulsive force was loaded at the center of the actomyosin ring, the force generated by actomyosin ring contraction was counter-balanced by the impulsive force.

Because the actomyosin ring contracts in a concentric fashion, from outside to inside, the force generated by the actomyosin ring contraction can be estimated from a relationship between the counter-balanced radius *R* and the incident laser pulse energy *L*. The impulsive force can be calibrated by using AFM as reported previously (Iino and Hosokawa, 2010). When the femtosecond laser is focused in the vicinity of the AFM cantilever, the total force *F*_0_ generated at the laser focal point is estimated from the bending movement of the cantilever. From the plot shown in Fig. S1A, the relationship between *L* and *F*_0_ can be expressed as:
(1)
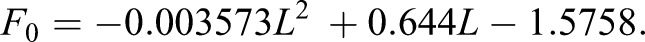
The total force *F*_0_ generated at the center of the actomyosin ring is calculated from *L* by Eqn 1.

**Fig. 3.**
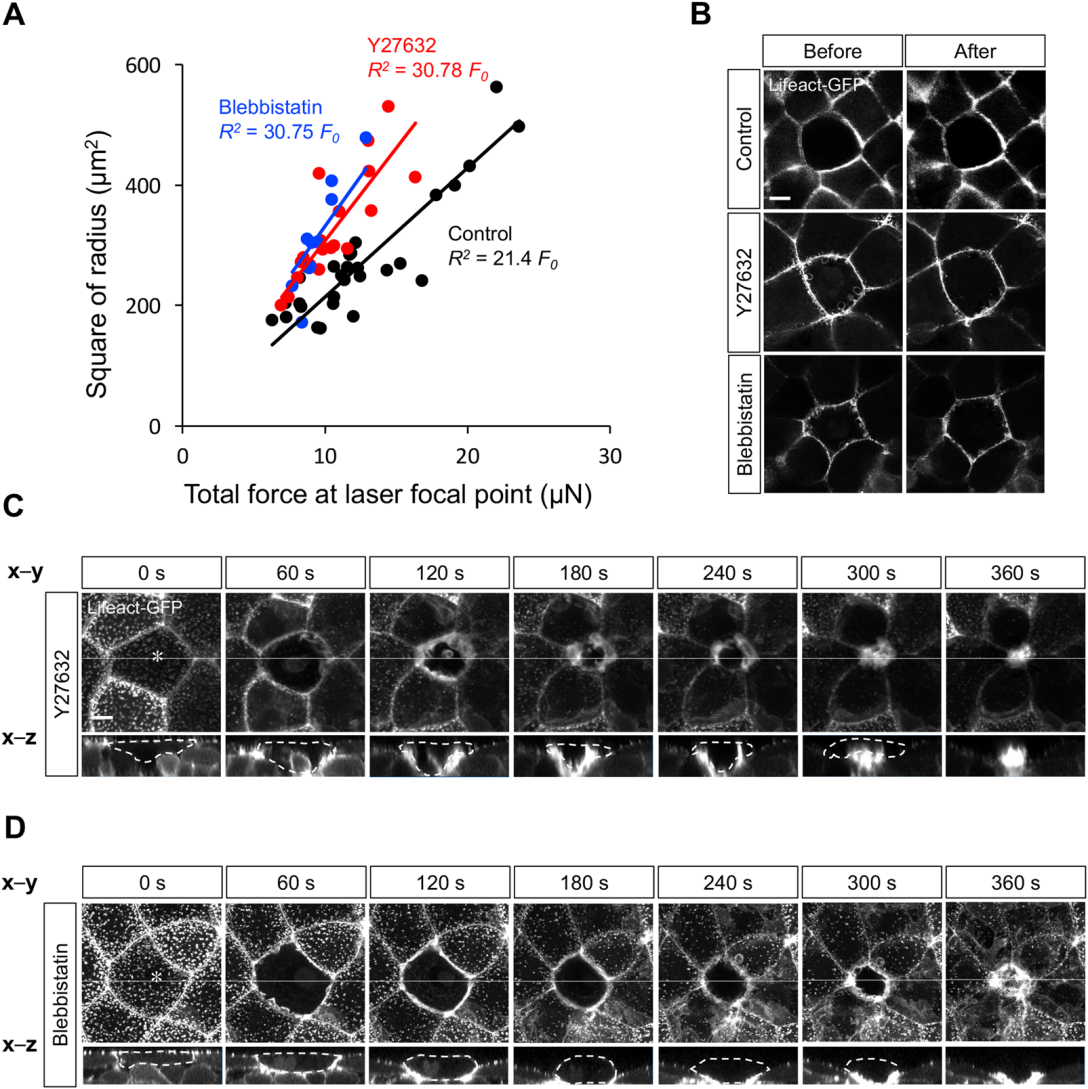
**Myosin II activity is required for force generation during cell extrusion.** (A) Square of radius *R*^2^ is proportional to total force *F*_0_ in Control (black dots), Y27632- (red dots), and Blebbistatin-treated embryos (blue dots) (correlation coefficient, *R*^2^=0.77, 0.68, and 0.70 in Control, Y27632-, and Blebbistatin-treated embryos). Magnitude of contractile force produced during cell extrusion in Control, Y27632-, or Blebbistatin-treated embryos was estimated, and is shown in Table 1. (B) Representative images of actomyosin ring before (left panels) and after (right panels) loading of impulsive force. Upper panels: Control embryo; middle panels: Y27632-treated embryo; lower panels: Blebbistatin-treated embryo. Upon treatment with 10 µM of Y27632 or 50 µM of Blebbistatin, the actomyosin ring was still formed. (C,D) Representative images of dynamics of F-actin during cell extrusion in a Lifeact–GFP-overexpressing embryo treated with Y27632 (C) or Blebbistatin (D). Still images were extracted from Movies 4 and 5. The x-z views of images (lower panels) were obtained by the cross-section at the white line in x-y views (upper panels). At time 0 s, the focal point marked by asterisks was irradiated with the femtosecond laser. Scale bars: 10 µm.

We investigated the relationship between the square of the ring radius *R*^2^ and *L* in Lifeact–GFP-overexpressing embryos, in which *L* varied from 10 to 60 nJ/pulse and *R*^2^ was evaluated as a function of *L* (Fig. S1B). When *L* was converted to *F*_0_ by Eqn 1, we observed a linear correlation between *R*^2^ and *F*_0_ (Fig. 3A, Control). This result clearly indicates that the impulsive force is counter-balanced by the contractile force of the actomyosin ring during laser irradiation.

From the linear correlation, we can estimate the pressure loaded on the actomyosin ring as a constant value. Assuming that *F*_0_ propagates spherically as a volume wave in the vicinity of the laser focal point, the pressure *P* at distance *R* from the laser focal point is expressed by:
(2)
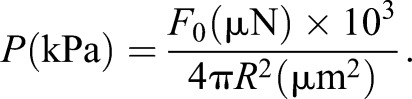
As the laser is focused at the center of the actomyosin ring, when the radius of the actomyosin ring is *R*, *P* corresponds to pressure loaded on the actomyosin ring. The data in Fig. 3A are least-squares fitted by:
(3)
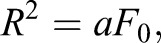
and the linear factor *a* is converted to the pressure *P* by:
(4)
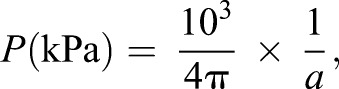
which is derived from Eqn 2. Accordingly, the force of contraction of the actomyosin ring is estimated to be 3.71 kPa (Table 1, Control). This represents the first *in vivo* measurement result of the mechanical force required for cell extrusion.

**Table 1.**
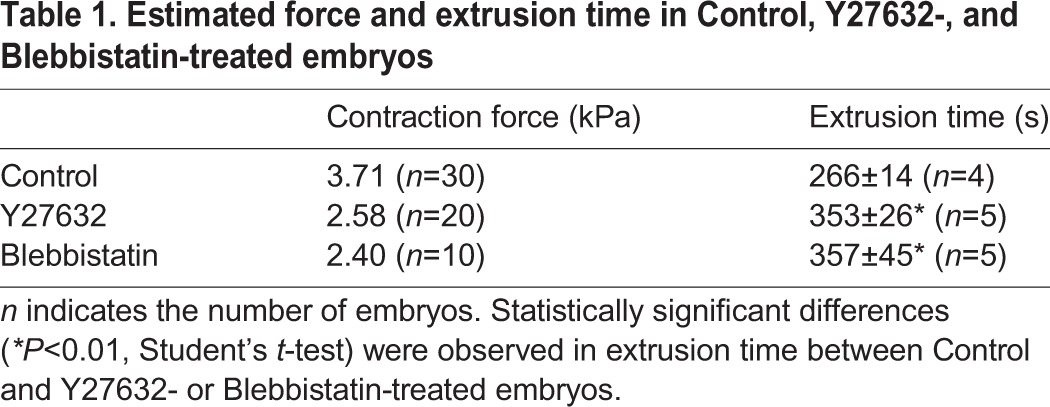
**Estimated force and extrusion time in Control, Y27632-, and Blebbistatin-treated embryos**

### Relative quantitative evaluation of contraction force generated by the actomyosin ring

Our quantitative analysis of the contraction force (this study) suggested that cell extrusion is driven by a force of relatively small magnitude produced by actomyosin ring contraction. To confirm this conclusion, we partially inhibited actomyosin contraction by treatment with Y27632 (a ROCK inhibitor) or Blebbistatin (a myosin II inhibitor) and investigated the effects on the mechanical dynamics of the actomyosin ring. We measured the mechanical force generated by actomyosin ring contraction in Y27632- or Blebbistatin-treated embryos by loading impulsive forces onto the ring (Fig. 3B). Under these conditions, the force was estimated to be 2.58 or 2.40 kPa, smaller than in the control (3.71 kPa) (Fig. 3A, Table 1). An actomyosin ring was still formed in the manipulated embryos following direct femtosecond laser irradiation, but cell extrusion was delayed for approximately 100 s (Fig. 3C,D, Table 1; Movies 4 and 5). These results suggest that the dynamics of cell extrusion are drastically affected by slight modulation of the contraction force. Taking all results together, we conclude that contraction of the actomyosin ring can generate a mechanical force on the order of kPa, and that this force of relatively small magnitude is capable of properly removing the targeted cell during cell extrusion in zebrafish.

## DISCUSSION

Based on technical advances in mechano-biology, mechanical forces generated by living cells have been measured using AFM, TFM, micropillar assay, and SCFS (Balaban et al., 2001; Krieg et al., 2008; Munevar et al., 2001; Stewart et al., 2011). However, in these systems, cells must be dissected from the tissues and organs of living organisms. Consequently, it has been difficult to measure a mechanical force under physiological conditions (e.g. within tissues or organs of living organisms). In this study, we sought to devise a system for measuring such forces during the process of cell extrusion in living zebrafish epithelia, and succeeded in measuring this force under physiological conditions. Our results revealed that a relatively small force generated by surrounding cells is sufficient to drive cell extrusion, highlighting the importance of mechanical regulation of this physiological phenomenon.

Using femotosecond laser-induced impulsive force, we estimated that a mechanical force on the order of kPa is generated during cell extrusion. Since nanosecond ultra-violet laser has been mainly used as a light source for the laser ablation (Behrndt et al., 2012; Campinho et al., 2013), nanosecond laser seems to be applicable for the force measurement during cell extrusion. However, in fact, nanosecond laser is not suitable for the force measurement because nanosecond laser is absorbed in the light pass and the absorbed light energy is mainly converted to heat, but not to impulsive force. Because no system has been developed that is capable of measuring mechanical force *in vivo*, it is very difficult to judge the accuracy of our measurement system. However, our estimates of the magnitude of this force are comparable to those of the traction force generated by cell–matrix interactions *in vitro* (Fu et al., 2010; Geiger et al., 2001). These findings thus support the accuracy of our measurement system.

In our system, we load a series of impulsive forces at the center of the actomyosin ring and estimate the force resulting from contraction of the actomyosin ring. Thus, we consider that actomyosin ring contraction produces extrusion force. However, actomyosin ring contraction is not the only source of the extrusion force: other force factors (e.g. circumferential contraction of the extruding cell, protrusive forces from surrounding cells and pushing forces from deep cell underneath) may also contribute to cell extrusion. Accordingly, in the near future, it would be of interest to investigate how the magnitude of the force is changed upon manipulation of other force generating processes by applying anisotropic tension for epithelial cells, and removing deep cells (Campinho et al., 2013; Morita et al., 2017) and/or upon loading of the impulsive force at different places, including cell protrusions.

Physiological phenomena, including epiboly, dorsal closure, cell competition, and wound healing, which occur in different tissues and organs in different species, use mechanisms similar to those of cell extrusion (Kajita et al., 2010; Schwayer et al., 2016). Due to the lack of appropriate experimental systems, their mechanical features have not yet been characterized. Therefore, our measurement system represents a powerful tool for measuring the forces involved in these physiological phenomena, and provides an understanding of how mechanical force contributes to the regulation of these phenomena. We are currently applying our system to studies of cell competition and wound healing in both cultured MDCK cells and zebrafish embryos, with the goal of elucidating the mechanical properties underlying these processes.

## MATERIAL AND METHODS

### Zebrafish experiments

Wild-type zebrafish were used in this study. All zebrafish experiments were performed with the approval of the Nara Institute of Science and Technology's Animal Studies Committee.

### Synthesis of mRNA and injection

pCS2-*Lifeact-GFP* and pCS2-*MRLC-GFP* [gifts from Drs Noriyuki Kinoshita (National Institute for Basic Biology, Japan) and Yasuyuki Fujita (The University of Hokkaido, Japan), respectively] were used as templates for mRNA synthesis. *Lifeact-GFP* and *MRLC-GFP* mRNAs were synthesized using the SP6 mMessage mMachine System (Thermo Fisher Scientific). *Lifeact-GFP* mRNA (100 pg) or Myosin II regulatory light chain-GFP (*MRLC-GFP*) mRNA (200 pg) were injected into the yolk of one-cell-stage zebrafish embryos, as described previously (Matsui et al., 2005).

### Inhibitor treatment

Injected embryos were developed until 5 h post-fertilization (hpf), treated with 10 µM Y27632 (Nacalai Tesque, Kyoto, Japan) or 50 µM Blebbistatin (Sigma-Aldrich) for 60 min, and then used for experiments. Non-treated or 0.1% DMSO (vehicle)-treated embryos were used as negative controls.

### Induction of cell extrusion in zebrafish embryonic epithelia and observation of dynamics of actomyosin

Injected embryos were developed at around 6 hpf, dechorionated, and mounted in the holes of a gel made with 1% low-melting-point agarose (Nacalai Tesque) on 35 mm glass bottom dishes (Matsunami, Osaka, Japan). A single shot of 800 nm laser pulse (15 nJ/pulse), generated by a titanium-sapphire femtosecond laser system (Solstice Ace, Spectra-Physics, California, USA), was focused through a 100×/1.25 objective lens (Olympus) into the center of epithelial cells located near the animal poles of embryos at 6 hpf. Dynamic changes of the actomyosin ring were observed with a confocal microscope (FV300, Olympus) for 5–10 min at 1–15 s intervals. *Z*-stack images of the embryos (8–17 planes at 1 or 2 μm intervals) were obtained. Actomyosin ring size (µm^2^) in each time point was measured by Image J software (NIH).

### Quantification of mechanical force generated by actomyosin ring contraction

Single shot of the femtosecond laser (15 nJ/pulse) was focused into the center of epithelial cell. Next, when the actomyosin ring was formed and started to contract (approximately 120 s after laser irradiation), a series of impulsive forces (10–60 nJ/pulse) was loaded (50 times at 1 s intervals) at the center of the actomyosin ring. Dynamic changes of the actomyosin ring were observed with a confocal microscope (FV300, Olympus) for 5 min at 1 s intervals. Actomyosin ring size (µm^2^) in each time point was measured by Image J. In addition, counter-balanced radius *R* (µm) of the ring was measured. Using the measurement results and Eqns 1–4, force generated by actomyosin ring contraction was estimated as described in the Results.

### Statistical analysis

Differences in means were analyzed by one-tailed Student's *t*-test. The results of *t*-tests were considered significant when *P*<0.01.
